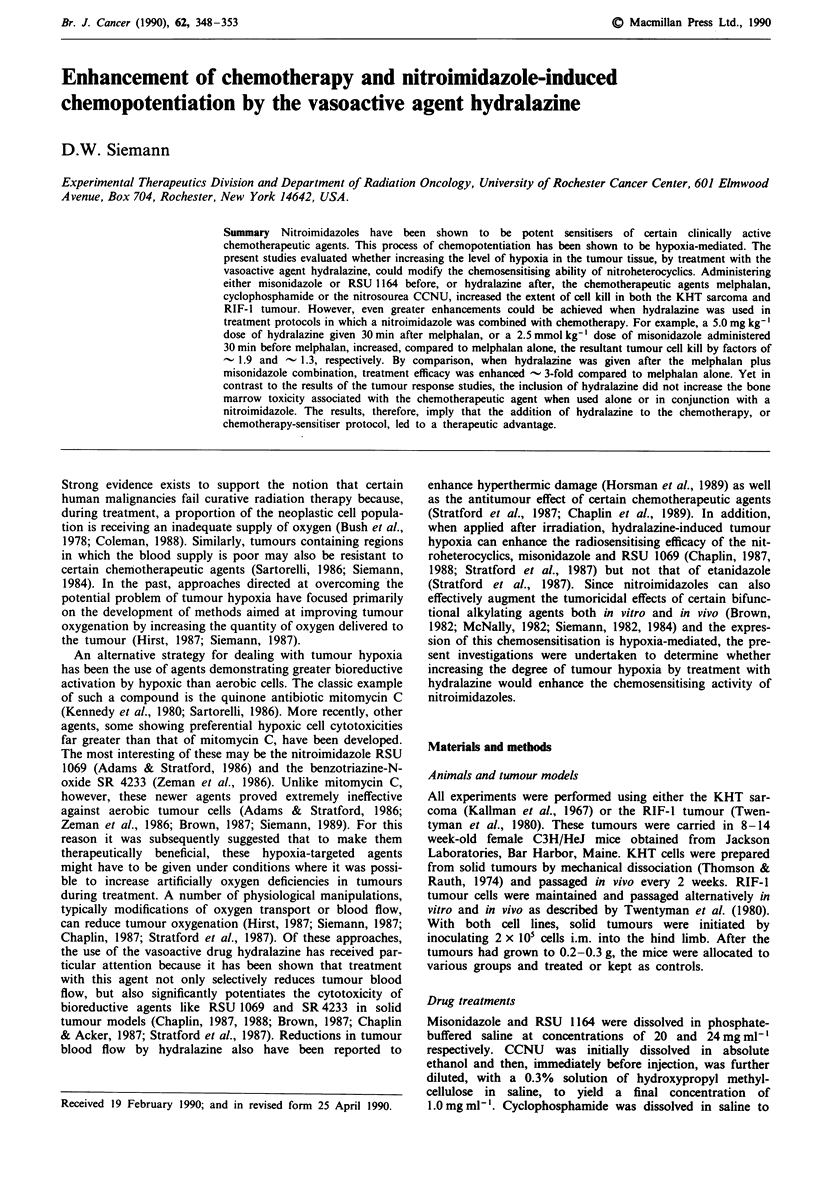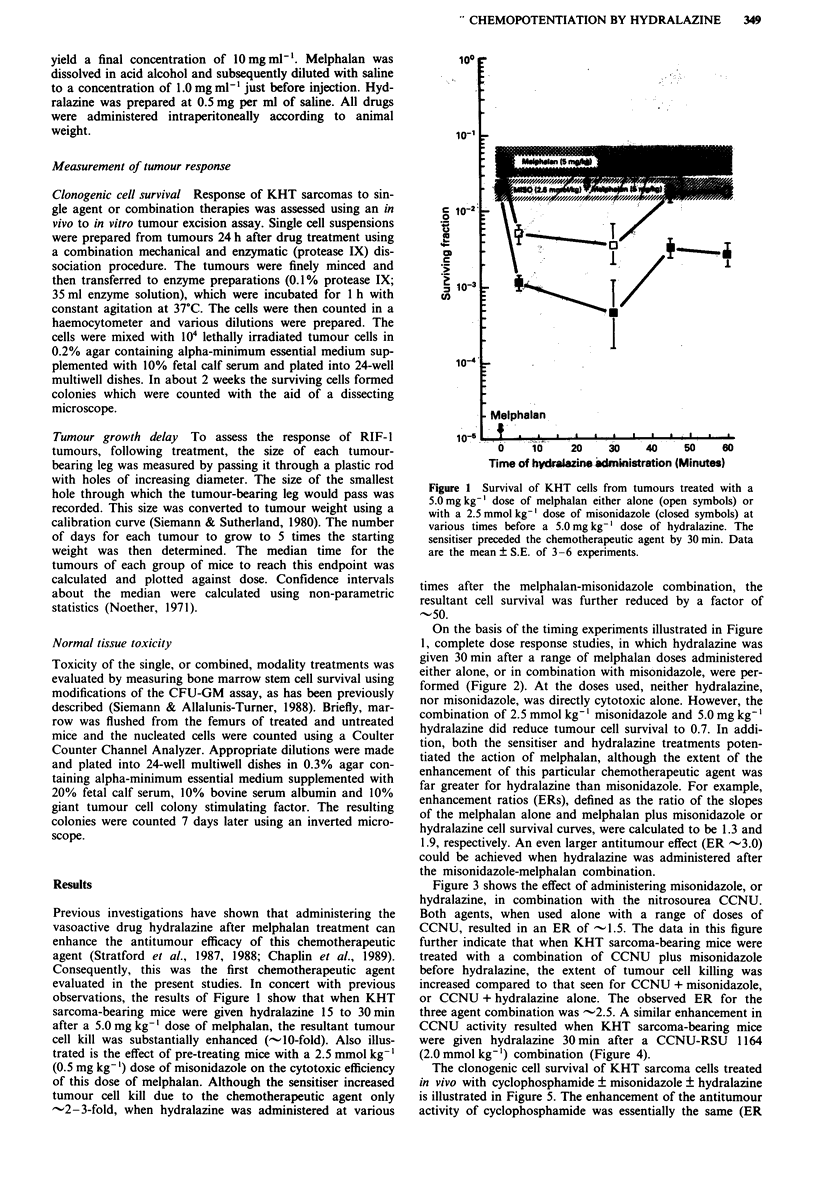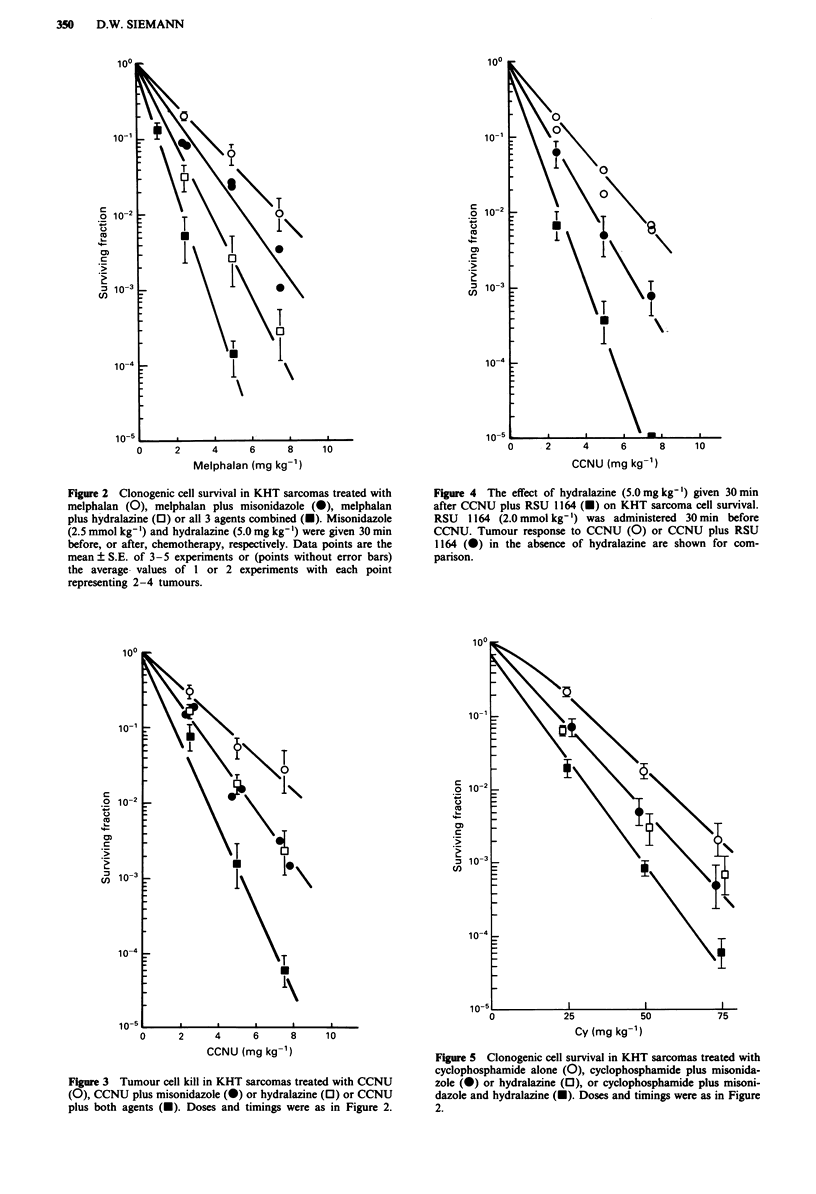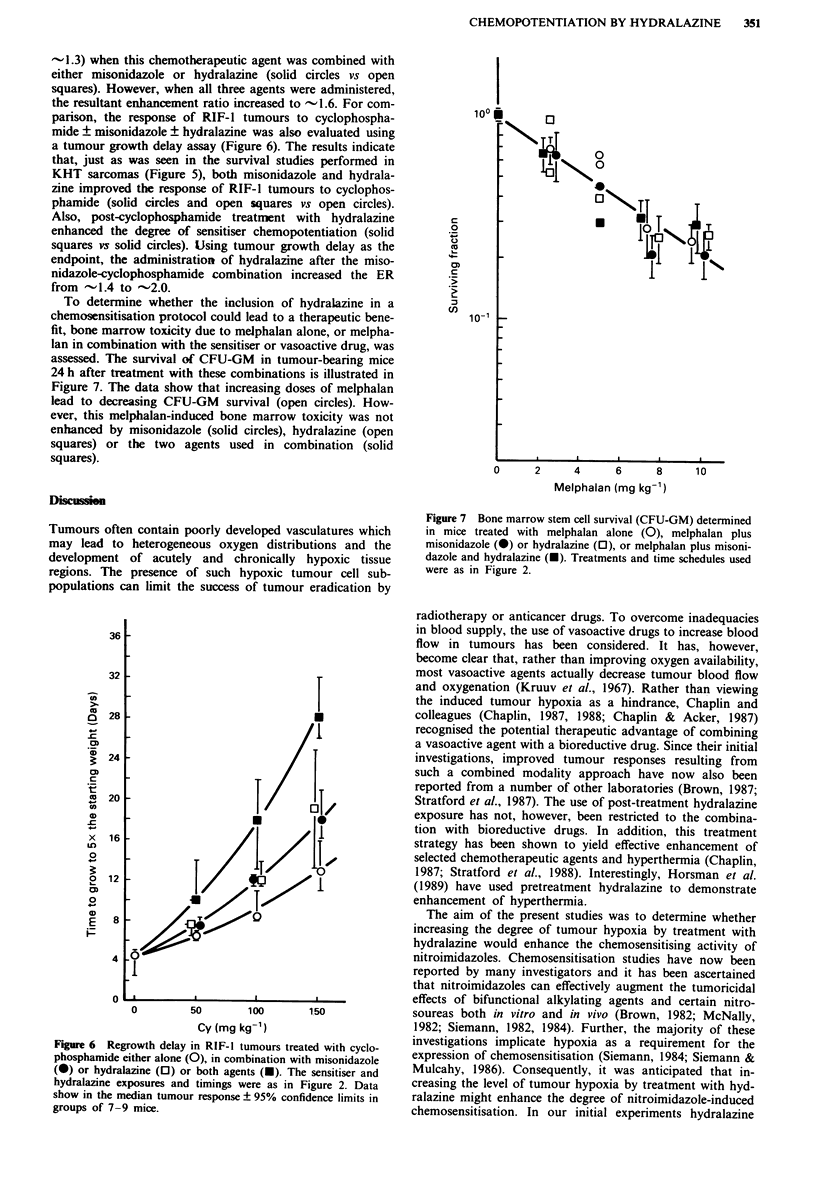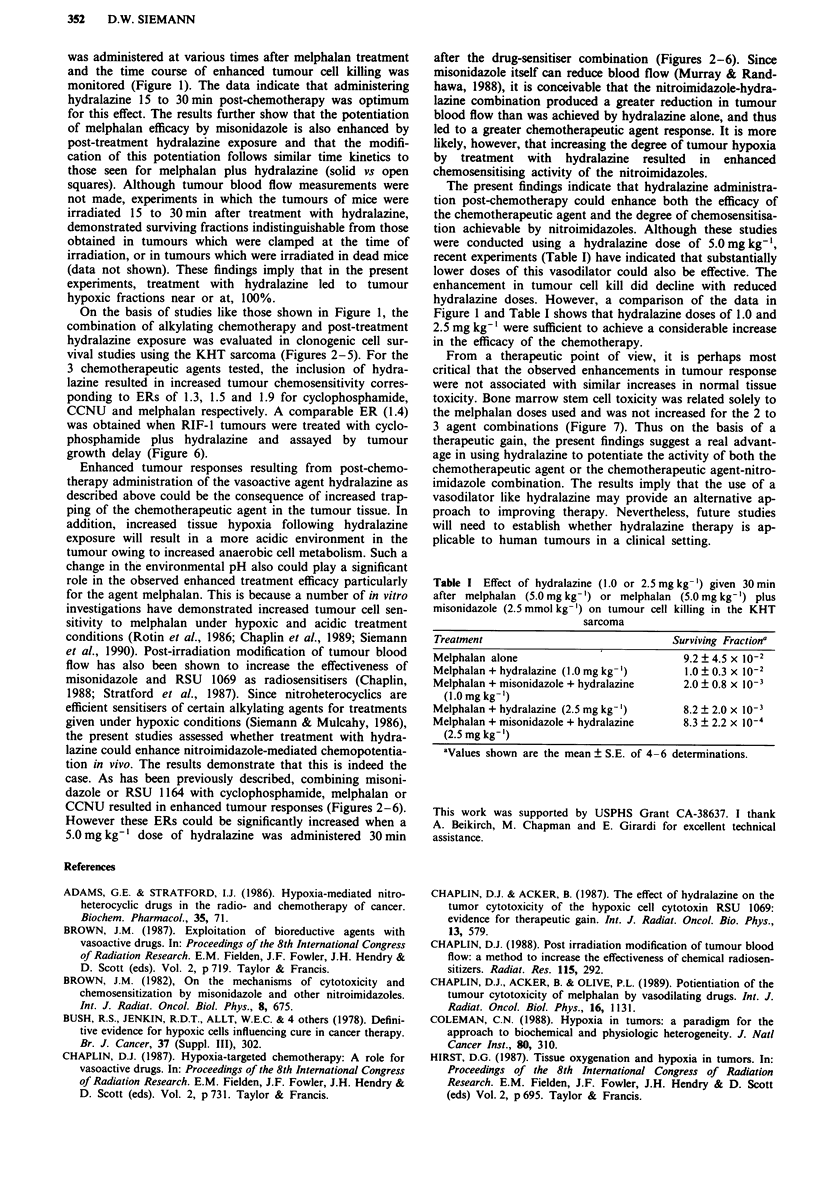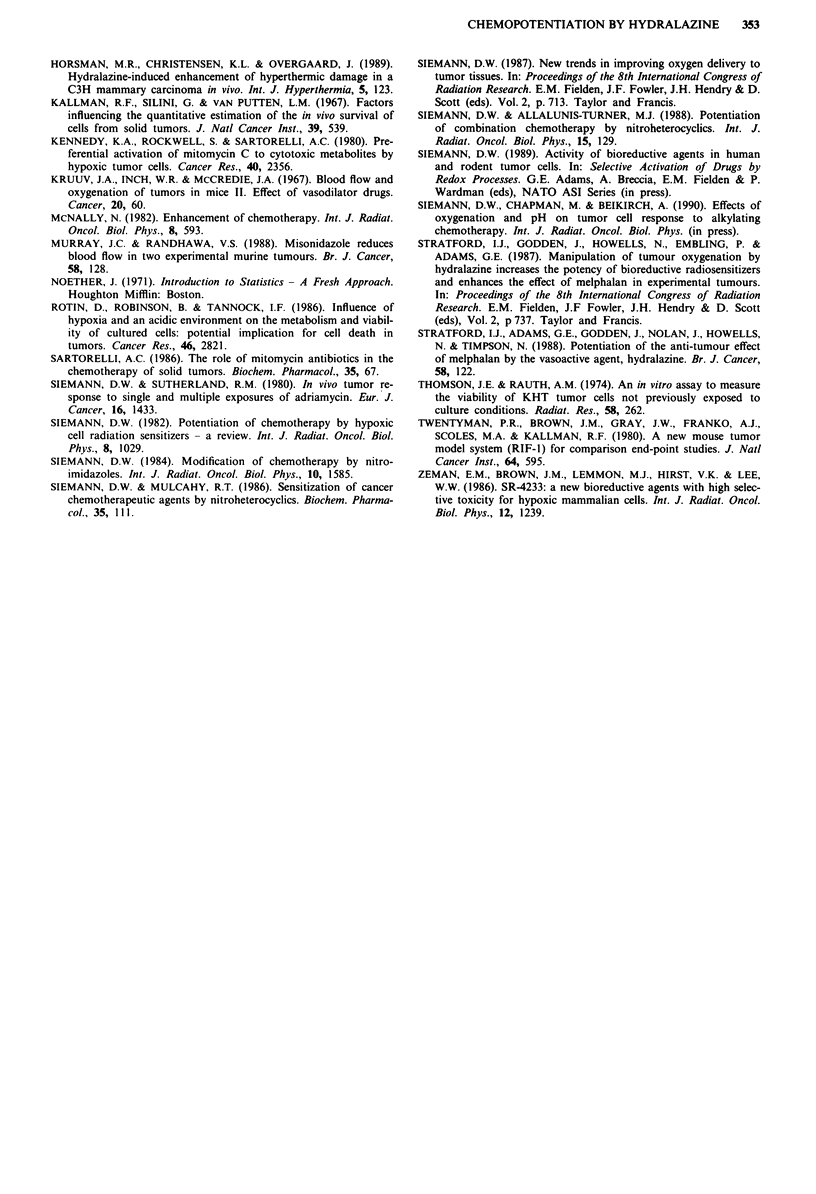# Enhancement of chemotherapy and nitroimidazole-induced chemopotentiation by the vasoactive agent hydralazine.

**DOI:** 10.1038/bjc.1990.295

**Published:** 1990-09

**Authors:** D. W. Siemann

**Affiliations:** Experimental Therapeutics Division, University of Rochester Cancer Center, New York 14642.

## Abstract

Nitroimidazoles have been shown to be potent sensitisers of certain clinically active chemotherapeutic agents. This process of chemopotentiation has been shown to be hypoxia-mediated. The present studies evaluated whether increasing the level of hypoxia in the tumour tissue, by treatment with the vasoactive agent hydralazine, could modify the chemosensitising ability of nitroheterocyclics. Administering either misonidazole or RSU 1164 before, or hydralazine after, the chemotherapeutic agents melphalan, cyclophosphamide or the nitrosourea CCNU, increased the extent of cell kill in both the KHT sarcoma and RIF-1 tumour. However, even greater enhancements could be achieved when hydralazine was used in treatment protocols in which a nitroimidazole was combined with chemotherapy. For example, a 5.0 mg kg-1 dose of hydralazine given 30 min after melphalan, or a 2.5 mmol kg-1 dose of misonidazole administered 30 min before melphalan, increased, compared to melphalan alone, the resultant tumour cell kill by factors of approximately 1.9 and approximately 1.3, respectively. By comparison, when hydralazine was given after the melphalan plus misonidazole combination, treatment efficacy was enhanced approximately 3-fold compared to melphalan alone. Yet in contrast to the results of the tumour response studies, the inclusion of hydralazine did not increase the bone marrow toxicity associated with the chemotherapeutic agent when used alone or in conjunction with a nitroimidazole. The results, therefore, imply that the addition of hydralazine to the chemotherapy, or chemotherapy-sensitiser protocol, led to a therapeutic advantage.


Br. J. Cancer (1990), 62, 348-353 © Macmillan Press Ltd., 1990

### Enhancement of chemotherapy and nitroimidazole-induced chemopotentiation by the vasoactive agent hydralazine

D.W. Siemann

*Experimental Therapeutics Division and Department of Radiation Oncology, University of Rochester Cancer Center, 601 Elmwood Avenue, Box 704, Rochester, New York 14642, USA.*

**Summary** Nitroimidazoles have been shown to be potent sensitisers of certain clinically active chemotherapeutic agents. This process of chemopotentiation has been shown to be hypoxia-mediated. The present studies evaluated whether increasing the level of hypoxia in the tumour tissue, by treatment with the vasoactive agent hydralazine, could modify the chemosensitising ability of nitroheterocyclics. Administering either misonidazole or RSU 1164 before, or hydralazine after, the chemotherapeutic agents melphalan, cyclophosphamide or the nitrosourea CCNU, increased the extent of cell kill in both the KHT sarcoma and RIF-1 tumour. However, even greater enhancements could be achieved when hydralazine was used in treatment protocols in which a nitroimidazole was combined with chemotherapy. For example, a 5.0 mg $kg^{-1}$ dose of hydralazine given 30 min after melphalan, or a 2.5 mmol $kg^{-1}$ dose of misonidazole administered 30 min before melphalan, increased, compared to melphalan alone, the resultant tumour cell kill by factors of ~ 1.9 and ~ 1.3, respectively. By comparison, when hydralazine was given after the melphalan plus misonidazole combination, treatment efficacy was enhanced ~ 3-fold compared to melphalan alone. Yet in contrast to the results of the tumour response studies, the inclusion of hydralazine did not increase the bone marrow toxicity associated with the chemotherapeutic agent when used alone or in conjunction with a nitroimidazole. The results, therefore, imply that the addition of hydralazine to the chemotherapy, or chemotherapy-sensitiser protocol, led to a therapeutic advantage.

Strong evidence exists to support the notion that certain human malignancies fail curative radiation therapy because, during treatment, a proportion of the neoplastic cell population is receiving an inadequate supply of oxygen (Bush *et al.*, 1978; Coleman, 1988). Similarly, tumours containing regions in which the blood supply is poor may also be resistant to certain chemotherapeutic agents (Sartorelli, 1986; Siemann, 1984). In the past, approaches directed at overcoming the potential problem of tumour hypoxia have focused primarily on the development of methods aimed at improving tumour oxygenation by increasing the quantity of oxygen delivered to the tumour (Hirst, 1987; Siemann, 1987).

An alternative strategy for dealing with tumour hypoxia has been the use of agents demonstrating greater bioreductive activation by hypoxic than aerobic cells. The classic example of such a compound is the quinone antibiotic mitomycin C (Kennedy *et al.*, 1980; Sartorelli, 1986). More recently, other agents, some showing preferential hypoxic cell cytotoxicities far greater than that of mitomycin C, have been developed. The most interesting of these may be the nitroimidazole RSU 1069 (Adams & Stratford, 1986) and the benzotriazine-N-oxide SR 4233 (Zeman *et al.*, 1986). Unlike mitomycin C, however, these newer agents proved extremely ineffective against aerobic tumour cells (Adams & Stratford, 1986; Zeman *et al.*, 1986; Brown, 1987; Siemann, 1989). For this reason it was subsequently suggested that to make them therapeutically beneficial, these hypoxia-targeted agents might have to be given under conditions where it was possible to increase artificially oxygen deficiencies in tumours during treatment. A number of physiological manipulations, typically modifications of oxygen transport or blood flow, can reduce tumour oxygenation (Hirst, 1987; Siemann, 1987; Chaplin, 1987; Stratford *et al.*, 1987). Of these approaches, the use of the vasoactive drug hydralazine has received particular attention because it has been shown that treatment with this agent not only selectively reduces tumour blood flow, but also significantly potentiates the cytotoxicity of bioreductive agents like RSU 1069 and SR 4233 in solid tumour models (Chaplin, 1987, 1988; Brown, 1987; Chaplin & Acker, 1987; Stratford *et al.*, 1987). Reductions in tumour blood flow by hydralazine also have been reported to enhance hyperthermic damage (Horsman *et al.*, 1989) as well as the antitumour effect of certain chemotherapeutic agents (Stratford *et al.*, 1987; Chaplin *et al.*, 1989). In addition, when applied after irradiation, hydralazine-induced tumour hypoxia can enhance the radiosensitising efficacy of the nitroheterocyclics, misonidazole and RSU 1069 (Chaplin, 1987, 1988; Stratford *et al.*, 1987) but not that of etanidazole (Stratford *et al.*, 1987). Since nitroimidazoles can also effectively augment the tumoricidal effects of certain bifunctional alkylating agents both *in vitro* and *in vivo* (Brown, 1982; McNally, 1982; Siemann, 1982, 1984) and the expression of this chemosensitisation is hypoxia-mediated, the present investigations were undertaken to determine whether increasing the degree of tumour hypoxia by treatment with hydralazine would enhance the chemosensitising activity of nitroimidazoles.

Received 19 February 1990; and in revised form 25 April 1990.

### Materials and methods

### *Animals and tumour models*

All experiments were performed using either the KHT sarcoma (Kallman *et al.*, 1967) or the RIF-1 tumour (Twentyman *et al.*, 1980). These tumours were carried in 8-14 week-old female C3H/HeJ mice obtained from Jackson Laboratories, Bar Harbor, Maine. KHT cells were prepared from solid tumours by mechanical dissociation (Thomson & Rauth, 1974) and passaged *in vivo* every 2 weeks. RIF-1 tumour cells were maintained and passaged alternatively *in vitro* and *in vivo* as described by Twentyman *et al.* (1980). With both cell lines, solid tumours were initiated by inoculating $2 \times 10^5$ cells i.m. into the hind limb. After the tumours had grown to 0.2-0.3 g, the mice were allocated to various groups and treated or kept as controls.

### *Drug treatments*

Misonidazole and RSU 1164 were dissolved in phosphate-buffered saline at concentrations of 20 and 24 mg $ml^{-1}$ respectively. CCNU was initially dissolved in absolute ethanol and then, immediately before injection, was further diluted, with a 0.3% solution of hydroxypropyl methylcellulose in saline, to yield a final concentration of 1.0 mg $ml^{-1}$. Cyclophosphamide was dissolved in saline to yield a final concentration of $10\,mg\,ml^{-1}$. Melphalan was dissolved in acid alcohol and subsequently diluted with saline to a concentration of $1.0\,mg\,ml^{-1}$ just before injection. Hydralazine was prepared at 0.5 mg per ml of saline. All drugs were administered intraperitoneally according to animal weight.

*Measurement of tumour response*

*Clonogenic cell survival* Response of KHT sarcomas to single agent or combination therapies was assessed using an *in vivo* to *in vitro* tumour excision assay. Single cell suspensions were prepared from tumours 24 h after drug treatment using a combination mechanical and enzymatic (protease IX) dissociation procedure. The tumours were finely minced and then transferred to enzyme preparations (0.1% protease IX; 35 ml enzyme solution), which were incubated for 1 h with constant agitation at 37°C. The cells were then counted in a haemocytometer and various dilutions were prepared. The cells were mixed with $10^4$ lethally irradiated tumour cells in 0.2% agar containing alpha-minimum essential medium supplemented with 10% fetal calf serum and plated into 24-well multiwell dishes. In about 2 weeks the surviving cells formed colonies which were counted with the aid of a dissecting microscope.

*Tumour growth delay* To assess the response of RIF-1 tumours, following treatment, the size of each tumour-bearing leg was measured by passing it through a plastic rod with holes of increasing diameter. The size of the smallest hole through which the tumour-bearing leg would pass was recorded. This size was converted to tumour weight using a calibration curve (Siemann & Sutherland, 1980). The number of days for each tumour to grow to 5 times the starting weight was then determined. The median time for the tumours of each group of mice to reach this endpoint was calculated and plotted against dose. Confidence intervals about the median were calculated using non-parametric statistics (Noether, 1971).

*Normal tissue toxicity*

Toxicity of the single, or combined, modality treatments was evaluated by measuring bone marrow stem cell survival using modifications of the CFU-GM assay, as has been previously described (Siemann & Allalunis-Turner, 1988). Briefly, marrow was flushed from the femurs of treated and untreated mice and the nucleated cells were counted using a Coulter Counter Channel Analyzer. Appropriate dilutions were made and plated into 24-well multiwell dishes in 0.3% agar containing alpha-minimum essential medium supplemented with 20% fetal calf serum, 10% bovine serum albumin and 10% giant tumour cell colony stimulating factor. The resulting colonies were counted 7 days later using an inverted microscope.

**Results**

Previous investigations have shown that administering the vasoactive drug hydralazine after melphalan treatment can enhance the antitumour efficacy of this chemotherapeutic agent (Stratford *et al.*, 1987, 1988; Chaplin *et al.*, 1989). Consequently, this was the first chemotherapeutic agent evaluated in the present studies. In concert with previous observations, the results of Figure 1 show that when KHT sarcoma-bearing mice were given hydralazine 15 to 30 min after a $5.0\,mg\,kg^{-1}$ dose of melphalan, the resultant tumour cell kill was substantially enhanced (~10-fold). Also illustrated is the effect of pre-treating mice with a $2.5\,mmol\,kg^{-1}$ ($0.5\,mg\,kg^{-1}$) dose of misonidazole on the cytotoxic efficiency of this dose of melphalan. Although the sensitiser increased tumour cell kill due to the chemotherapeutic agent only ~2–3-fold, when hydralazine was administered at various times after the melphalan-misonidazole combination, the resultant cell survival was further reduced by a factor of ~50.

**Figure 1** Survival of KHT cells from tumours treated with a $5.0\,mg\,kg^{-1}$ dose of melphalan either alone (open symbols) or with a $2.5\,mmol\,kg^{-1}$ dose of misonidazole (closed symbols) at various times before a $5.0\,mg\,kg^{-1}$ dose of hydralazine. The sensitiser preceded the chemotherapeutic agent by 30 min. Data are the mean ± S.E. of 3–6 experiments.

On the basis of the timing experiments illustrated in Figure 1, complete dose response studies, in which hydralazine was given 30 min after a range of melphalan doses administered either alone, or in combination with misonidazole, were performed (Figure 2). At the doses used, neither hydralazine, nor misonidazole, was directly cytotoxic alone. However, the combination of $2.5\,mmol\,kg^{-1}$ misonidazole and $5.0\,mg\,kg^{-1}$ hydralazine did reduce tumour cell survival to 0.7. In addition, both the sensitiser and hydralazine treatments potentiated the action of melphalan, although the extent of the enhancement of this particular chemotherapeutic agent was far greater for hydralazine than misonidazole. For example, enhancement ratios (ERs), defined as the ratio of the slopes of the melphalan alone and melphalan plus misonidazole or hydralazine cell survival curves, were calculated to be 1.3 and 1.9, respectively. An even larger antitumour effect (ER ~3.0) could be achieved when hydralazine was administered after the misonidazole-melphalan combination.

Figure 3 shows the effect of administering misonidazole, or hydralazine, in combination with the nitrosourea CCNU. Both agents, when used alone with a range of doses of CCNU, resulted in an ER of ~1.5. The data in this figure further indicate that when KHT sarcoma-bearing mice were treated with a combination of CCNU plus misonidazole before hydralazine, the extent of tumour cell killing was increased compared to that seen for CCNU + misonidazole, or CCNU + hydralazine alone. The observed ER for the three agent combination was ~2.5. A similar enhancement in CCNU activity resulted when KHT sarcoma-bearing mice were given hydralazine 30 min after a CCNU-RSU 1164 ($2.0\,mmol\,kg^{-1}$) combination (Figure 4).

The clonogenic cell survival of KHT sarcoma cells treated *in vivo* with cyclophosphamide ± misonidazole ± hydralazine is illustrated in Figure 5. The enhancement of the antitumour activity of cyclophosphamide was essentially the same (ER

**Figure 2** Clonogenic cell survival in KHT sarcomas treated with melphalan (○), melphalan plus misonidazole (●), melphalan plus hydralazine (□) or all 3 agents combined (■). Misonidazole (2.5 mmol $kg^{-1}$) and hydralazine (5.0 mg $kg^{-1}$) were given 30 min before, or after, chemotherapy, respectively. Data points are the mean ± S.E. of 3–5 experiments or (points without error bars) the average values of 1 or 2 experiments with each point representing 2–4 tumours.

**Figure 4** The effect of hydralazine (5.0 mg $kg^{-1}$) given 30 min after CCNU plus RSU 1164 (■) on KHT sarcoma cell survival. RSU 1164 (2.0 mmol $kg^{-1}$) was administered 30 min before CCNU. Tumour response to CCNU (○) or CCNU plus RSU 1164 (●) in the absence of hydralazine are shown for comparison.

**Figure 3** Tumour cell kill in KHT sarcomas treated with CCNU (○), CCNU plus misonidazole (●) or hydralazine (□) or CCNU plus both agents (■). Doses and timings were as in Figure 2.

**Figure 5** Clonogenic cell survival in KHT sarcomas treated with cyclophosphamide alone (○), cyclophosphamide plus misonidazole (●) or hydralazine (□), or cyclophosphamide plus misonidazole and hydralazine (■). Doses and timings were as in Figure 2.

~1.3) when this chemotherapeutic agent was combined with either misonidazole or hydralazine (solid circles *vs* open squares). However, when all three agents were administered, the resultant enhancement ratio increased to ~1.6. For comparison, the response of RIF-1 tumours to cyclophosphamide ± misonidazole ± hydralazine was also evaluated using a tumour growth delay assay (Figure 6). The results indicate that, just as was seen in the survival studies performed in KHT sarcomas (Figure 5), both misonidazole and hydralazine improved the response of RIF-1 tumours to cyclophosphamide (solid circles and open squares *vs* open circles). Also, post-cyclophosphamide treatment with hydralazine enhanced the degree of sensitiser chemopotentiation (solid squares *vs* solid circles). Using tumour growth delay as the endpoint, the administration of hydralazine after the misonidazole-cyclophosphamide combination increased the ER from ~1.4 to ~2.0.

To determine whether the inclusion of hydralazine in a chemosensitisation protocol could lead to a therapeutic benefit, bone marrow toxicity due to melphalan alone, or melphalan in combination with the sensitiser or vasoactive drug, was assessed. The survival of CFU-GM in tumour-bearing mice 24 h after treatment with these combinations is illustrated in Figure 7. The data show that increasing doses of melphalan lead to decreasing CFU-GM survival (open circles). However, this melphalan-induced bone marrow toxicity was not enhanced by misonidazole (solid circles), hydralazine (open squares) or the two agents used in combination (solid squares).

**Figure 7** Bone marrow stem cell survival (CFU-GM) determined in mice treated with melphalan alone (○), melphalan plus misonidazole (●) or hydralazine (□), or melphalan plus misonidazole and hydralazine (■). Treatments and time schedules used were as in Figure 2.

### Discussion

Tumours often contain poorly developed vasculatures which may lead to heterogeneous oxygen distributions and the development of acutely and chronically hypoxic tissue regions. The presence of such hypoxic tumour cell subpopulations can limit the success of tumour eradication by radiotherapy or anticancer drugs. To overcome inadequacies in blood supply, the use of vasoactive drugs to increase blood flow in tumours has been considered. It has, however, become clear that, rather than improving oxygen availability, most vasoactive agents actually decrease tumour blood flow and oxygenation (Kruuv *et al.*, 1967). Rather than viewing the induced tumour hypoxia as a hindrance, Chaplin and colleagues (Chaplin, 1987, 1988; Chaplin & Acker, 1987) recognised the potential therapeutic advantage of combining a vasoactive agent with a bioreductive drug. Since their initial investigations, improved tumour responses resulting from such a combined modality approach have now also been reported from a number of other laboratories (Brown, 1987; Stratford *et al.*, 1987). The use of post-treatment hydralazine exposure has not, however, been restricted to the combination with bioreductive drugs. In addition, this treatment strategy has been shown to yield effective enhancement of selected chemotherapeutic agents and hyperthermia (Chaplin, 1987; Stratford *et al.*, 1988). Interestingly, Horsman *et al.* (1989) have used pretreatment hydralazine to demonstrate enhancement of hyperthermia.

The aim of the present studies was to determine whether increasing the degree of tumour hypoxia by treatment with hydralazine would enhance the chemosensitising activity of nitroimidazoles. Chemosensitisation studies have now been reported by many investigators and it has been ascertained that nitroimidazoles can effectively augment the tumoricidal effects of bifunctional alkylating agents and certain nitrosoureas both *in vitro* and *in vivo* (Brown, 1982; McNally, 1982; Siemann, 1982, 1984). Further, the majority of these investigations implicate hypoxia as a requirement for the expression of chemosensitisation (Siemann, 1984; Siemann & Mulcahy, 1986). Consequently, it was anticipated that increasing the level of tumour hypoxia by treatment with hydralazine might enhance the degree of nitroimidazole-induced chemosensitisation. In our initial experiments hydralazine

**Figure 6** Regrowth delay in RIF-1 tumours treated with cyclophosphamide either alone (○), in combination with misonidazole (●) or hydralazine (□) or both agents (■). The sensitiser and hydralazine exposures and timings were as in Figure 2. Data show in the median tumour response ± 95% confidence limits in groups of 7–9 mice.

was administered at various times after melphalan treatment and the time course of enhanced tumour cell killing was monitored (Figure 1). The data indicate that administering hydralazine 15 to 30 min post-chemotherapy was optimum for this effect. The results further show that the potentiation of melphalan efficacy by misonidazole is also enhanced by post-treatment hydralazine exposure and that the modification of this potentiation follows similar time kinetics to those seen for melphalan plus hydralazine (solid *vs* open squares). Although tumour blood flow measurements were not made, experiments in which the tumours of mice were irradiated 15 to 30 min after treatment with hydralazine, demonstrated surviving fractions indistinguishable from those obtained in tumours which were clamped at the time of irradiation, or in tumours which were irradiated in dead mice (data not shown). These findings imply that in the present experiments, treatment with hydralazine led to tumour hypoxic fractions near or at, 100%.

On the basis of studies like those shown in Figure 1, the combination of alkylating chemotherapy and post-treatment hydralazine exposure was evaluated in clonogenic cell survival studies using the KHT sarcoma (Figures 2–5). For the 3 chemotherapeutic agents tested, the inclusion of hydralazine resulted in increased tumour chemosensitivity corresponding to ERs of 1.3, 1.5 and 1.9 for cyclophosphamide, CCNU and melphalan respectively. A comparable ER (1.4) was obtained when RIF-1 tumours were treated with cyclophosphamide plus hydralazine and assayed by tumour growth delay (Figure 6).

Enhanced tumour responses resulting from post-chemotherapy administration of the vasoactive agent hydralazine as described above could be the consequence of increased trapping of the chemotherapeutic agent in the tumour tissue. In addition, increased tissue hypoxia following hydralazine exposure will result in a more acidic environment in the tumour owing to increased anaerobic cell metabolism. Such a change in the environmental pH also could play a significant role in the observed enhanced treatment efficacy particularly for the agent melphalan. This is because a number of *in vitro* investigations have demonstrated increased tumour cell sensitivity to melphalan under hypoxic and acidic treatment conditions (Rotin *et al.*, 1986; Chaplin *et al.*, 1989; Siemann *et al.*, 1990). Post-irradiation modification of tumour blood flow has also been shown to increase the effectiveness of misonidazole and RSU 1069 as radiosensitisers (Chaplin, 1988; Stratford *et al.*, 1987). Since nitroheterocyclics are efficient sensitisers of certain alkylating agents for treatments given under hypoxic conditions (Siemann & Mulcahy, 1986), the present studies assessed whether treatment with hydralazine could enhance nitroimidazole-mediated chemopotentiation *in vivo*. The results demonstrate that this is indeed the case. As has been previously described, combining misonidazole or RSU 1164 with cyclophosphamide, melphalan or CCNU resulted in enhanced tumour responses (Figures 2–6). However these ERs could be significantly increased when a 5.0 mg kg$^{-1}$ dose of hydralazine was administered 30 min after the drug-sensitiser combination (Figures 2–6). Since misonidazole itself can reduce blood flow (Murray & Randhawa, 1988), it is conceivable that the nitroimidazole-hydralazine combination produced a greater reduction in tumour blood flow than was achieved by hydralazine alone, and thus led to a greater chemotherapeutic agent response. It is more likely, however, that increasing the degree of tumour hypoxia by treatment with hydralazine resulted in enhanced chemosensitising activity of the nitroimidazoles.

The present findings indicate that hydralazine administration post-chemotherapy could enhance both the efficacy of the chemotherapeutic agent and the degree of chemosensitisation achievable by nitroimidazoles. Although these studies were conducted using a hydralazine dose of 5.0 mg kg$^{-1}$, recent experiments (Table I) have indicated that substantially lower doses of this vasodilator could also be effective. The enhancement in tumour cell kill did decline with reduced hydralazine doses. However, a comparison of the data in Figure 1 and Table I shows that hydralazine doses of 1.0 and 2.5 mg kg$^{-1}$ were sufficient to achieve a considerable increase in the efficacy of the chemotherapy.

From a therapeutic point of view, it is perhaps most critical that the observed enhancements in tumour response were not associated with similar increases in normal tissue toxicity. Bone marrow stem cell toxicity was related solely to the melphalan doses used and was not increased for the 2 to 3 agent combinations (Figure 7). Thus on the basis of a therapeutic gain, the present findings suggest a real advantage in using hydralazine to potentiate the activity of both the chemotherapeutic agent or the chemotherapeutic agent-nitroimidazole combination. The results imply that the use of a vasodilator like hydralazine may provide an alternative approach to improving therapy. Nevertheless, future studies will need to establish whether hydralazine therapy is applicable to human tumours in a clinical setting.

**Table I** Effect of hydralazine (1.0 or 2.5 mg kg$^{-1}$) given 30 min after melphalan (5.0 mg kg$^{-1}$) or melphalan (5.0 mg kg$^{-1}$) plus misonidazole (2.5 mmol kg$^{-1}$) on tumour cell killing in the KHT sarcoma

| *Treatment* | *Surviving Fraction*[a] |
|---|---|
| Melphalan alone | $9.2 \pm 4.5 \times 10^{-2}$ |
| Melphalan + hydralazine (1.0 mg kg$^{-1}$) | $1.0 \pm 0.3 \times 10^{-2}$ |
| Melphalan + misonidazole + hydralazine (1.0 mg kg$^{-1}$) | $2.0 \pm 0.8 \times 10^{-3}$ |
| Melphalan + hydralazine (2.5 mg kg$^{-1}$) | $8.2 \pm 2.0 \times 10^{-3}$ |
| Melphalan + misonidazole + hydralazine (2.5 mg kg$^{-1}$) | $8.3 \pm 2.2 \times 10^{-4}$ |

[a]Values shown are the mean ± S.E. of 4–6 determinations.

This work was supported by USPHS Grant CA-38637. I thank A. Beikirch, M. Chapman and E. Girardi for excellent technical assistance.

### References

ADAMS, G.E. & STRATFORD, I.J. (1986). Hypoxia-mediated nitroheterocyclic drugs in the radio- and chemotherapy of cancer. *Biochem. Pharmacol.*, **35**, 71.

BROWN, J.M. (1987). Exploitation of bioreductive agents with vasoactive drugs. In: *Proceedings of the 8th International Congress of Radiation Research*. E.M. Fielden, J.F. Fowler, J.H. Hendry & D. Scott (eds). Vol. 2, p 719. Taylor & Francis.

BROWN, J.M. (1982), On the mechanisms of cytotoxicity and chemosensitization by misonidazole and other nitroimidazoles. *Int. J. Radiat. Oncol. Biol. Phys.*, **8**, 675.

BUSH, R.S., JENKIN, R.D.T., ALLT, W.E.C. & 4 others (1978). Definitive evidence for hypoxic cells influencing cure in cancer therapy. *Br. J. Cancer*, **37** (Suppl. III), 302.

CHAPLIN, D.J. (1987). Hypoxia-targeted chemotherapy: A role for vasoactive drugs. In: *Proceedings of the 8th International Congress of Radiation Research*. E.M. Fielden, J.F. Fowler, J.H. Hendry & D. Scott (eds). Vol. 2, p 731. Taylor & Francis.

CHAPLIN, D.J. & ACKER, B. (1987). The effect of hydralazine on the tumor cytotoxicity of the hypoxic cell cytotoxin RSU 1069: evidence for therapeutic gain. *Int. J. Radiat. Oncol. Bio. Phys.*, **13**, 579.

CHAPLIN, D.J. (1988). Post irradiation modification of tumour blood flow: a method to increase the effectiveness of chemical radiosensitizers. *Radiat. Res.* **115**, 292.

CHAPLIN, D.J., ACKER, B. & OLIVE, P.L. (1989). Potientiation of the tumour cytotoxicity of melphalan by vasodilating drugs. *Int. J. Radiat. Oncol. Biol. Phys.*, **16**, 1131.

COLEMAN, C.N. (1988). Hypoxia in tumors: a paradigm for the approach to biochemical and physiologic heterogeneity. *J. Natl Cancer Inst.*, **80**, 310.

HIRST, D.G. (1987). Tissue oxygenation and hypoxia in tumors. In: *Proceedings of the 8th International Congress of Radiation Research*. E.M. Fielden, J.F. Fowler, J.H. Hendry & D. Scott (eds) Vol. 2, p 695. Taylor & Francis.

HORSMAN, M.R., CHRISTENSEN, K.L. & OVERGAARD, J. (1989). Hydralazine-induced enhancement of hyperthermic damage in a C3H mammary carcinoma *in vivo*. *Int. J. Hyperthermia*, **5**, 123.

KALLMAN, R.F., SILINI, G. & VAN PUTTEN, L.M. (1967). Factors influencing the quantitative estimation of the *in vivo* survival of cells from solid tumors. *J. Natl Cancer Inst.*, **39**, 539.

KENNEDY, K.A., ROCKWELL, S. & SARTORELLI, A.C. (1980). Preferential activation of mitomycin C to cytotoxic metabolites by hypoxic tumor cells. *Cancer Res.*, **40**, 2356.

KRUUV, J.A., INCH, W.R. & MCCREDIE, J.A. (1967). Blood flow and oxygenation of tumors in mice II. Effect of vasodilator drugs. *Cancer*, **20**, 60.

MCNALLY, N. (1982). Enhancement of chemotherapy. *Int. J. Radiat. Oncol. Biol. Phys.*, **8**, 593.

MURRAY, J.C. & RANDHAWA, V.S. (1988). Misonidazole reduces blood flow in two experimental murine tumours. *Br. J. Cancer*, **58**, 128.

NOETHER, J. (1971). *Introduction to Statistics – A Fresh Approach*. Houghton Mifflin: Boston.

ROTIN, D., ROBINSON, B. & TANNOCK, I.F. (1986). Influence of hypoxia and an acidic environment on the metabolism and viability of cultured cells: potential implication for cell death in tumors. *Cancer Res.*, **46**, 2821.

SARTORELLI, A.C. (1986). The role of mitomycin antibiotics in the chemotherapy of solid tumors. *Biochem. Pharmacol.*, **35**, 67.

SIEMANN, D.W. & SUTHERLAND, R.M. (1980). *In vivo* tumor response to single and multiple exposures of adriamycin. *Eur. J. Cancer*, **16**, 1433.

SIEMANN, D.W. (1982). Potentiation of chemotherapy by hypoxic cell radiation sensitizers – a review. *Int. J. Radiat. Oncol. Biol. Phys.*, **8**, 1029.

SIEMANN, D.W. (1984). Modification of chemotherapy by nitroimidazoles. *Int. J. Radiat. Oncol. Biol. Phys.*, **10**, 1585.

SIEMANN, D.W. & MULCAHY, R.T. (1986). Sensitization of cancer chemotherapeutic agents by nitroheterocyclics. *Biochem. Pharmacol.*, **35**, 111.

SIEMANN, D.W. (1987). New trends in improving oxygen delivery to tumor tissues. In: *Proceedings of the 8th International Congress of Radiation Research*. E.M. Fielden, J.F. Fowler, J.H. Hendry & D. Scott (eds). Vol. 2, p. 713. Taylor and Francis.

SIEMANN, D.W. & ALLALUNIS-TURNER, M.J. (1988). Potentiation of combination chemotherapy by nitroheterocyclics. *Int. J. Radiat. Oncol. Biol. Phys.*, **15**, 129.

SIEMANN, D.W. (1989). Activity of bioreductive agents in human and rodent tumor cells. In: *Selective Activation of Drugs by Redox Processes*. G.E. Adams, A. Breccia, E.M. Fielden & P. Wardman (eds), NATO ASI Series (in press).

SIEMANN, D.W., CHAPMAN, M. & BEIKIRCH, A. (1990). Effects of oxygenation and pH on tumor cell response to alkylating chemotherapy. *Int. J. Radiat. Oncol. Biol. Phys.* (in press).

STRATFORD, I.J., GODDEN, J., HOWELLS, N., EMBLING, P. & ADAMS, G.E. (1987). Manipulation of tumour oxygenation by hydralazine increases the potency of bioreductive radiosensitizers and enhances the effect of melphalan in experimental tumours. In: *Proceedings of the 8th International Congress of Radiation Research*. E.M. Fielden, J.F Fowler, J.H. Hendry & D. Scott (eds), Vol. 2, p 737. Taylor and Francis.

STRATFORD, I.J., ADAMS, G.E., GODDEN, J., NOLAN, J., HOWELLS, N. & TIMPSON, N. (1988). Potentiation of the anti-tumour effect of melphalan by the vasoactive agent, hydralazine. *Br. J. Cancer*, **58**, 122.

THOMSON, J.E. & RAUTH, A.M. (1974). An *in vitro* assay to measure the viability of KHT tumor cells not previously exposed to culture conditions. *Radiat. Res.*, **58**, 262.

TWENTYMAN, P.R., BROWN, J.M., GRAY, J.W., FRANKO, A.J., SCOLES, M.A. & KALLMAN, R.F. (1980). A new mouse tumor model system (RIF-1) for comparison end-point studies. *J. Natl Cancer Inst.*, **64**, 595.

ZEMAN, E.M., BROWN, J.M., LEMMON, M.J., HIRST, V.K. & LEE, W.W. (1986). SR-4233: a new bioreductive agents with high selective toxicity for hypoxic mammalian cells. *Int. J. Radiat. Oncol. Biol. Phys.*, **12**, 1239.